# MiR-503 suppresses cell proliferation and invasion of gastric cancer by targeting HMGA2 and inactivating WNT signaling pathway

**DOI:** 10.1186/s12935-019-0875-1

**Published:** 2019-06-14

**Authors:** Wenjing Li, Jun Li, Hong Mu, Meiqi Guo, Haixia Deng

**Affiliations:** 1 Clinical Laboratory, Tianjin First Central Hospital, No. 24 Fukang Road, Nankai District, Tianjin, China; 2 Hematology Oncology Center, Beijing Children’s Hospital, Capital Medical University, National Center for Children’s Health, Beijing, 100045 China

**Keywords:** Gastric cancer, miR-503, HMGA2, Cell proliferation, Cell invasion

## Abstract

**Background:**

Abnormal expression of microRNAs (miRNAs) is related to human carcinogenesis. Although previous studies have shown that miR-503 expression in gastric cancer (GC) is downregulated, however, the underlying molecular mechanism for miR-503 involved in gastric cancer development is still largely unknown.

**Methods:**

The relative expression of miR-503 in GC tissues and adjacent normal tissues was examined using quantitative real-time reverse transcription polymerase chain reaction (qRT-PCR) analyses. In vitro, cell proliferation and invasion were evaluated by using CCK8, cell colony and transwell invasion assays. In vivo, xenograft tumor model was constructed to assess miR-503 expression whether affects tumor growth or not. Luciferase reporter assay, qRT-PCR and western blot assay were used to demonstrate HMGA2 is a target of miR-503.

**Results:**

We demonstrated that miR-503 expression was significantly downregulated in GC tissues and cell lines compared to adjacent normal tissues and normal gastric mucosa cell lines, respectively. Lower miR-503 expression associated with tumor size, lymph node metastasis, and predicted a poor overall survival (OS) time in GC patients. Subsequently, in vitro, gain-function and loss-function assays confirmed that miR-503 overexpression significantly suppressed GC cell proliferation, colony formation and cell invasion, while decreased miR-503 expression had an adverse effect in GC cells. Furthermore, we found that miR-503 specifically targeted the 3′-UTR regions of HMGA2 mRNA and suppressed its protein expression. Overexpression of HMGA2 could reverse the miR-503 mediated inhibition of GC cell proliferation and invasion. In vivo, miR-503 overexpression dramatically reduced tumor growth. Moreover, we demonstrated that miR-503 suppressed WNT/β-catenin signaling by elevating GSK-3β and p-β-catenin expression, but decreased p-GSK-3β and β-catenin expression in GC cells.

**Conclusion:**

These results provide that miR-503 expression acts as a predictor for GC prognosis and may have a potential application in GC therapy.

## Background

Gastric cancer (GC) is one of most common gastrointestinal malignancy and the second leading cause of cancer-related death worldwide. In China, there are nearly 460,000 new cases of the disease and more than 350,000 deaths each year [1, 2]. Patients who are often diagnosed at an advanced stage represent a poor 5-year overall survival (OS) rate due to tumor metastasis and recurrence [3, 4]. Thus, to investigate novel molecular mechanisms that be involved in GC development and explore novel methods for GC treatment is required.

MicroRNAs (miRNAs) can bind to the 3′ untranslated regions (UTRs) of their target mRNA to regulate gene expression at the posttranscription levels [5]. MiRNAs exert a wide variety of fundamental biological processes in GC development including cell proliferation, invasion, and metastasis [6]. Some of miRNAs are identified as potential prognostic biomarkers or therapeutic targets of GC [7, 8]. Deregulation of miR-503 expression contributes to the development of some tumors including gastric cancer. For example, downregulation of miR-503 expression predicates advanced mythological features and poor prognosis in patients with NSCLC [9]. Chong et al. found that microRNA-503 acts as a tumor suppressor in osteosarcoma by targeting L1CAM [10]. In hepatocellular carcinoma, miR-503 regulates tumor metastatic function through Rho guanine nucleotide exchanger factor 19 [11]. In gastric cancer, Peng et al. showed that microRNA-503 could inhibit gastric cancer cell growth and epithelial-to-mesenchymal transition process [12]. Other studies have revealed that miR-503 regulates cisplatin resistance of human gastric cancer cell lines by targeting IGF1R and BCL2 [13]. In spite of the broad involvement of miR-503 inhibiting tumorigenesis in various cancers including GC, the underling molecular mechanisms of miR-503 in GC remain little known.

In the study, we confirmed that miR-503 expression was downregulated in gastric cancer tissues and cell lines, respectively. Lower miR-503 expression predicted a poor prognosis in patients with GC. In vitro, miR-503 overexpression suppressed cell proliferation, migration and invasion in vitro. In vivo, miR-503 overexpression reduced tumor growth. Furthermore, we demonstrated that miR-503 inhibited cell proliferation and invasion by targeting HMGA2 and inactivated WNT signaling pathway in GC. Therefore, these results indicated that miR-503 may be a potential target of GC treatment.

## Materials and methods

### Patients and clinical tissue samples

A total of 46 patients who underwent GC radical surgical resection used in the study at the Tianjin First Central Hospital from February 2009 to November 2012. Tissue samples were immediately frozen in liquid nitrogen and stored at − 80 °C until RNA analysis. The patients (ranging from 30 to 72 years) had a median age of 55.45 years. Patients who received any treatment prior to surgery were excluded in the study. The clinical date of all subjects is shown in Table 1. Written Informed consent was obtained from all patients and the study was approved by the ethical committee of the Tianjin First Central Hospital.

**Table 1:** Correlation of miR-503 expression with clinicopathologic parameters in 46 GC patients

Clinicopathologic parameter	Total (n = 46)	MiR-503 expression level		*P*-value
		Lower (n = 24)	Higher (n = 22)	
Age (years)				0.211
≤ 55	27	12	15	
> 55	19	12	7	
Gender				0.829
Male	30	16	14	
Female	16	8	8	
Tumor size				0.008*
< 3	24	8	16	
≥ 3	22	16	6	
Histological grade				0.686
High and middle	30	15	15	
Low	16	9	7	
Lymph node metastasis				0.017*
No	25	9	16	
Yes	21	15	6	
TNM stage				0.460
I/II	31	15	16	
III/IV	15	9	6	
Local invasion				0.143
T1, T2	22	9	13	
T3, T4	24	15	9	

^*^P < 0.05

### Cell lines culture

The immortalized normal gastric mucosal epithelial cell line (GES-1) and human GC cell lines MKN-45, BGC-823, SGC-7901, MKN-28, and AGS were purchased from Shanghai Institute of Cell Biology (Shanghai, China). Cells were cultured in RPMI 1640 (Gibco; Thermo Fisher Scientific, Inc., Waltham, MA, USA) and supplemented with 10% fetal bovine serum (FBS) at 37 °C in a humidified atmosphere with 5% CO_2_.

### Transfection of oligonucleotides or plasmids

The miR-503 mimic, miR-negative control (miR-NC), or miR-503 inhibitor oligonucleotides were chemically synthesized and purchased from Ribobio (Guangzhou, China). Cells were plated in 6-well plates and transfected with 100 nM oligonucleotides (miR-503 mimic, miR-negative control, or miR-503 inhibitor) using Lipofectamine™ 2000 reagent following manufacturer’s instructions (Invitrogen, Carlsbad, CA, USA). Full-length HMGA2 complementary DNA (cDNA) were chemically synthesized and inserted into pcDNA3.1 vector (Invitrogen) to generate HMGA2 expression vector pcDNA3.1-HMGA2. Stabilized miRNAs (miR-503 agomir and NC agomir) were purchased from RiboBio (Guangzhou, China).

### RNA extraction and quantitative reverse transcription polymerase chain reaction (qRT-PCR)

GC tissues and cells were used to isolate RNA using Trizol Reagent (TAKAKA, Dalian, China) according to the manufacturer’s guidelines. Total RNA was reverse-transcribed using a TaqMan MicroRNA Reverse Transcription Kit (Applied Biosystems, Foster City, CA, USA). The mRNA expression was evaluated using SYBR Green qPCR Super Mix Kit (TAKALA, Dalian, China) on an ABI Prism® 7500 Sequence Detection System (Applied Biosystems). Fold change of miR-503 or was calculated by the equation 2^−ΔΔCt^ methods. The primer sequences were as follows: GAPDH or endogenous U6 small nuclear RNA (U6 snRNA) was used as control for mRNA or miRNA expression, respectively. The relative prime sequences were as follow: miR-503-forward: 5′-CCTATTTCCCATGATTCCTTCATA-3′, miR-503-reverse: 5′-GTAATACGGTTATCCACGCG-3′ HMGA2-forward, 5′-CAAGTTGTTCAGAAGAAGCCTGC-3′, HMGA2-reverse, 5′-CATGGCAATACAGAATAAGTGGTCAC-3′; GAPDH-forward, 5′-GGTCTCCTCTGACTTCAACA-3′, GAPDH-reverse, 5′-GTGAGGGTCTCTCTCTTCCT-3′.

### Cell proliferation assay

GC cells viability was measured using Cell Counting Kit-8 (Dojindo, Kumamoto, Japan) according to the manufacturer’s instructions. The MKN-45 and SGC-7901 cells (2000 cells/well) transfected with miR-503 mimic, miR-negative control, or miR-503 inhibitor were seeded in 96-well plates and cultured at 37 °C in a humidified atmosphere with 5% CO_2_. Then, cell viability was detected every 24 h until 96 h. The cell viability was detected and optical density value was 450 nm using an automatic multi-well spectrophotometer (Bio-Rad Laboratories, Inc., Hercules, CA, USA).

### Cell colony formation assay

MKN-45 or SGC-7901 cells (1 × 10^3^ cells/well) transfected with miR-503 mimic, miR-negative control, or miR-503 inhibitor were plated in 6-well plates and cultured at 37 °C in a humidified atmosphere with 5% CO_2_ for 14 days. Colonies were fixed using 100% methanol, stained with 0.1% crystal violet. Cell colonies were then counted and analyzed.

### Cell invasion assay

Cell invasion assays were performed using transwell chambers with 8 µm pore size membranes (BD Biosciences, San Jose, CA, USA). MKN-45 or SGC-7901 cells were resuspended with 300 µL free FBS culture medium and added on the upper chambers. A 500 µL medium containing 10% FBS was added to the lower chambers. Cells were cultured at 37 °C in a humidified atmosphere with 5% CO_2_. Then cells were cultured for 48 h, invasive cells on the lower chambers were fixed with 4% paraformaldehyde for 15 min and stained with 0.1% crystal violet for 15 min. Cells were counted in five random fields under a light microscope.

### Western blot analysis

The protein extraction was detected using RAPA buffer (KeyGen Biotech Co., Ltd, Nanjing, China). Equal proteins (30 µg) were separated on 10–12% discontinuous sodium dodecyl sulfate-polyacrylamide gels (SDS-PAGE) by electrophoresis and then electrophoretically transferred to polyvinylidene difluoride (PVDF) membranes. The membranes then were blocked with 5% milk and incubated at 4 °C overnight with primary antibodies including HMGA2 (Santa Cruz, CA, USA), β-catenin (Santa Cruz, CA, USA), GSK-3β (Santa Cruz, CA, USA), p-β-catenin (Santa Cruz, CA, USA), p-GSK-3β (Santa Cruz, CA, USA) and GAPDH (abcam). Then the membranes were incubated with secondary antibodies for 2 h at room temperature. The western blot bands were detected using enhanced chemiluminescence (ECL) reagents (Pierce, Rockford, IL, USA).

### Luciferase reporter assay

The wild-type (WT) HMGA2-3′UTR containing the putative miR-503 binding site or mutant HMGA2-3′UTR (MUT) (from GCUGCU to CGACGA) was cloned into the SpeI and MluI sites of the pMIR-Report luciferase vector (Applied Biosystems). MKN-45 cells were transfected with 50 ng per well wild-type or mutant type 3′UTR of HMGA2 luciferase vectors and 100 nM miR-503 mimic or miR-NC oligonucleotides. After transfection at 48 h, cells were lysated using Lysis Buffer (Promega) and luciferase activity was detected by using the Dual Luciferase Reporter Assay (Promega).

### Xenograft tumor model

MKN45 cells were stably transfected agomiR-control and agomiR-503 plasmid. BALB/c mice (female and 4 weeks old) were subcutaneously injected using stably transfected agomiR-control and agomiR-503 plasmids MKN-45 cells (1 × 10^6^ cells) into the left flank. Tumor growth was monitored every 7 days. Tumor volume was monitored by measuring the length and width, the tumor volume = (length × width^2^)/2. At 28 days after injection, mice were sacrificed and tumors were collected and the tumor weight was measured for analysis.

### Statistical analysis

Statistical analysis in the study was analyzed by using SPSS software (version 22.0; SPSS Inc). The Student *t* test was used to compare the differences between groups from at least three or more experiments. A two-tailed P-value of less than 0.05 was considered statistically significant.

## Results

### MiR-503 expression is downregulated in gastric tissues and cells

**Fig. 1:**
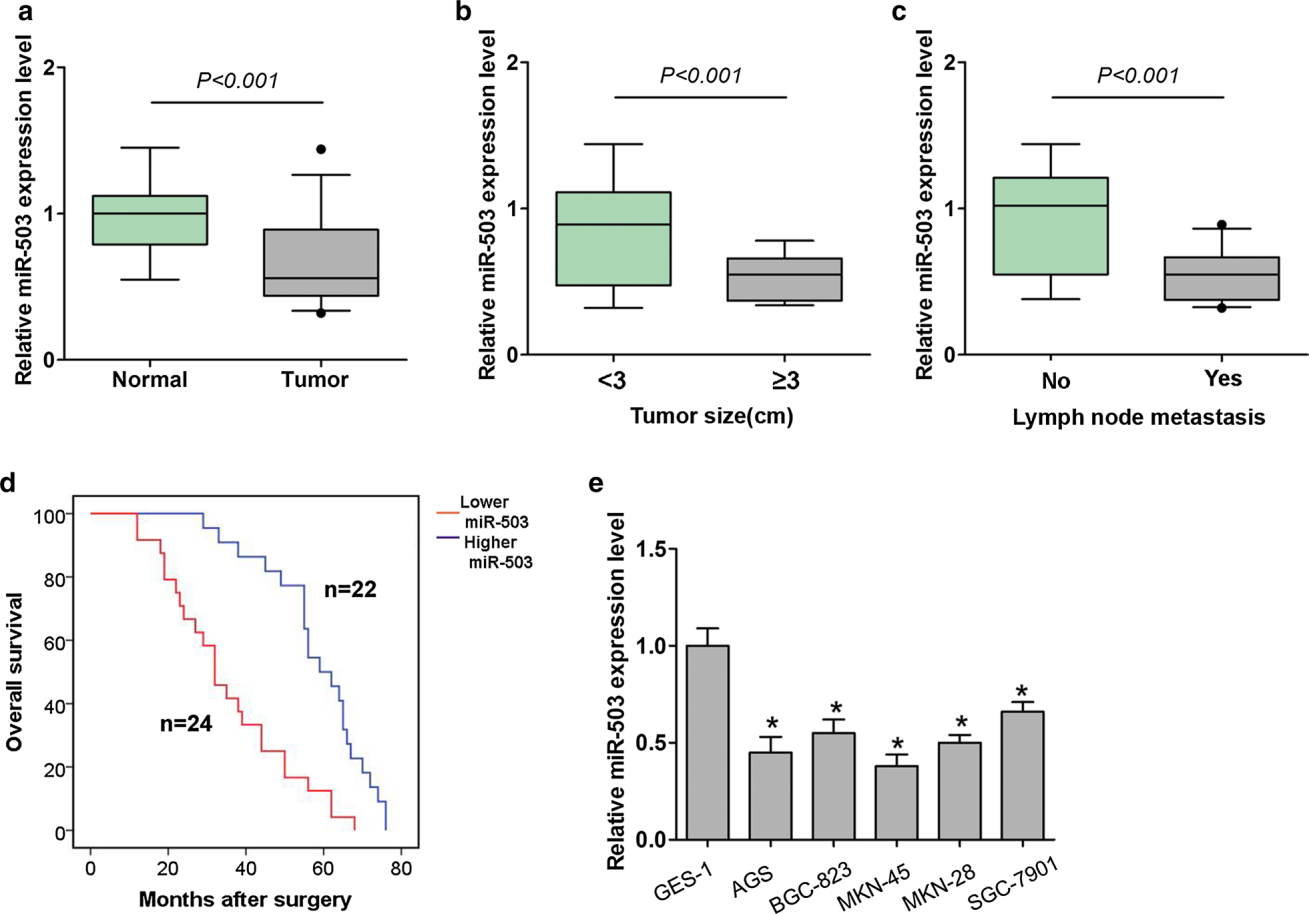
MiR-503 expression is downregulated in gastric tissues and cells. **a** The expression levels of miR-503 in 46 GC tissues and adjacent normal tissues were investigated and qRT-PCR analyses. **b** Association between miR-503 expression and tumor size and **c** lymph node metastasis in patients. **d** Kaplan–Meier analysis and log rank test showed that GC patients with lower miR-503 expression level (n = 24) predicted poorer OS rate than those patients with higher miR-503 expression level (n = 22). **e** The expression levels of miR-503 was detected in an immortalized normal gastric mucosal epithelial cell line (GES-1) and human GC cell lines MKN-45, BGC-823, SGC-7901, MKN-28, and AGS using qRT-PCR. All values are presented as mean ± SD, *P < 0.05

To validate the association between miR-503 expression and GC, we compared the mRNA expression levels in gastric cancer tissues and corresponding adjacent normal tissues using qRT-PCR. As represented in Fig. 1a, miR-503 expression levels were significantly downregulated in GC tissues compared to normal tissues. Also, the expression levels of miR-503 were reduced in GC tissues with large tumor size ( ≥ 3) and lymph node metastasis of GC patients (Fig. 1b, c, Table 1). Moreover, GC patients with lower miR-503 expression level (n = 24) predicted poorer OS rate than those patients with higher miR-503 expression level (n = 22) (Log rank = 12.05, *P* < 0.05, Fig. 1d). In addition, we analyzed the miR-503 expression in GES-1 cell and five GC cell lines. The results of qRT-PCR analyses indicated miR-503 expression was lower in GC cells compared to GES-1 cells (Fig. 1e). Thus, these results implied that miR-503 expression was downregulated in GC tissues and cells, and lower miR-503 expression predicted a poor prognosis in patients with GC.

### Effects of miR-503 expression on cell proliferation and invasion of GC

**Fig. 2:**
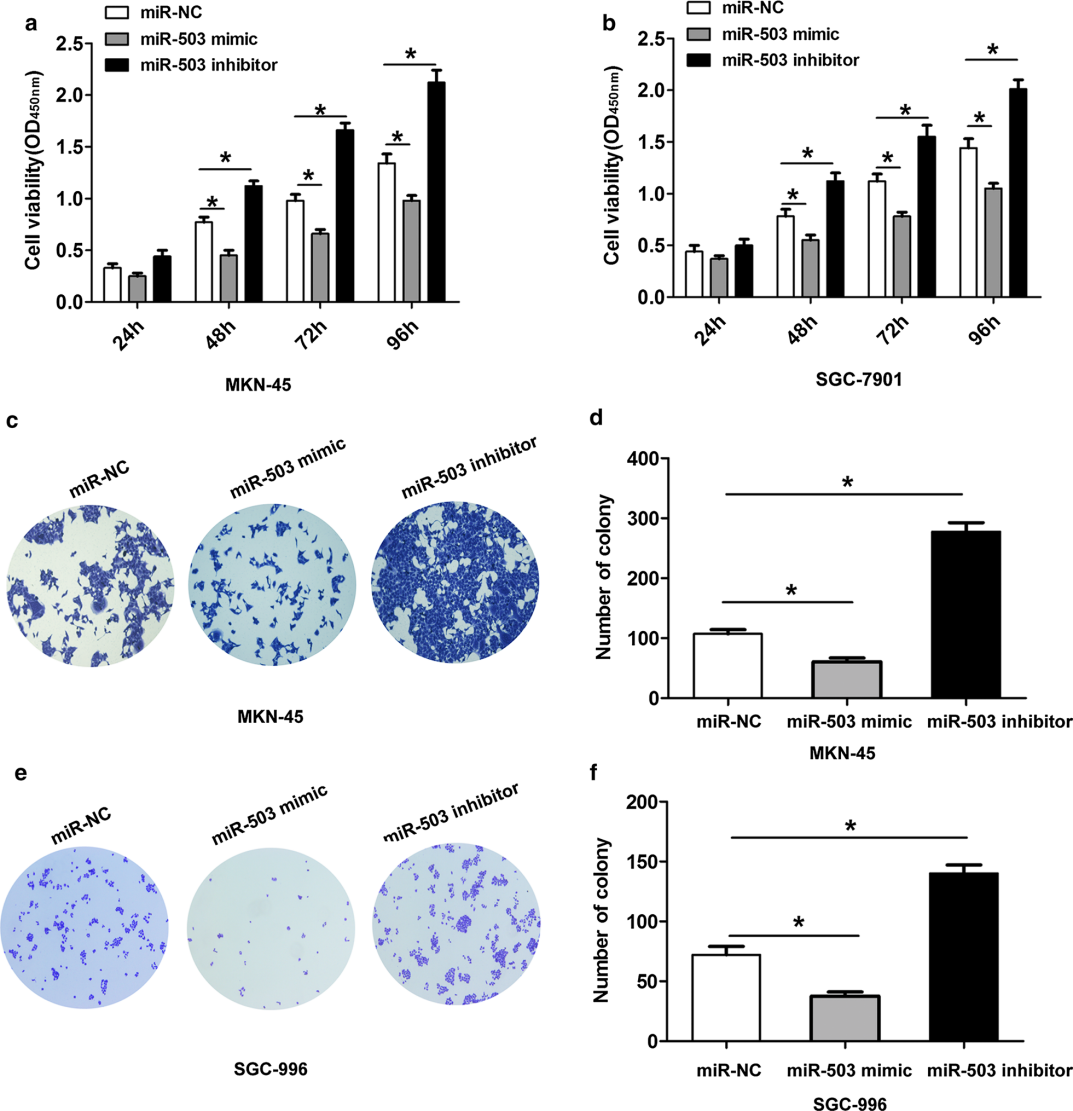
Effects of miR-503 expression on cell proliferation of GC. **a**, **b** Cell viability was showed at 24 h, 48 h, 72 h and 96 h after cell transfected with miR-NC, miR-503 mimic or miR-503 inhibitor in MKN-45 or SGC-7901 cells. **c**–**f** Cell colony formation was showed at 14 days after cell transfected with miR-NC, miR-503 mimic or miR-503 inhibitor in MKN-45 or SGC-7901 cells. All values are presented as mean ± SD, *P < 0.05

**Fig. 3:**
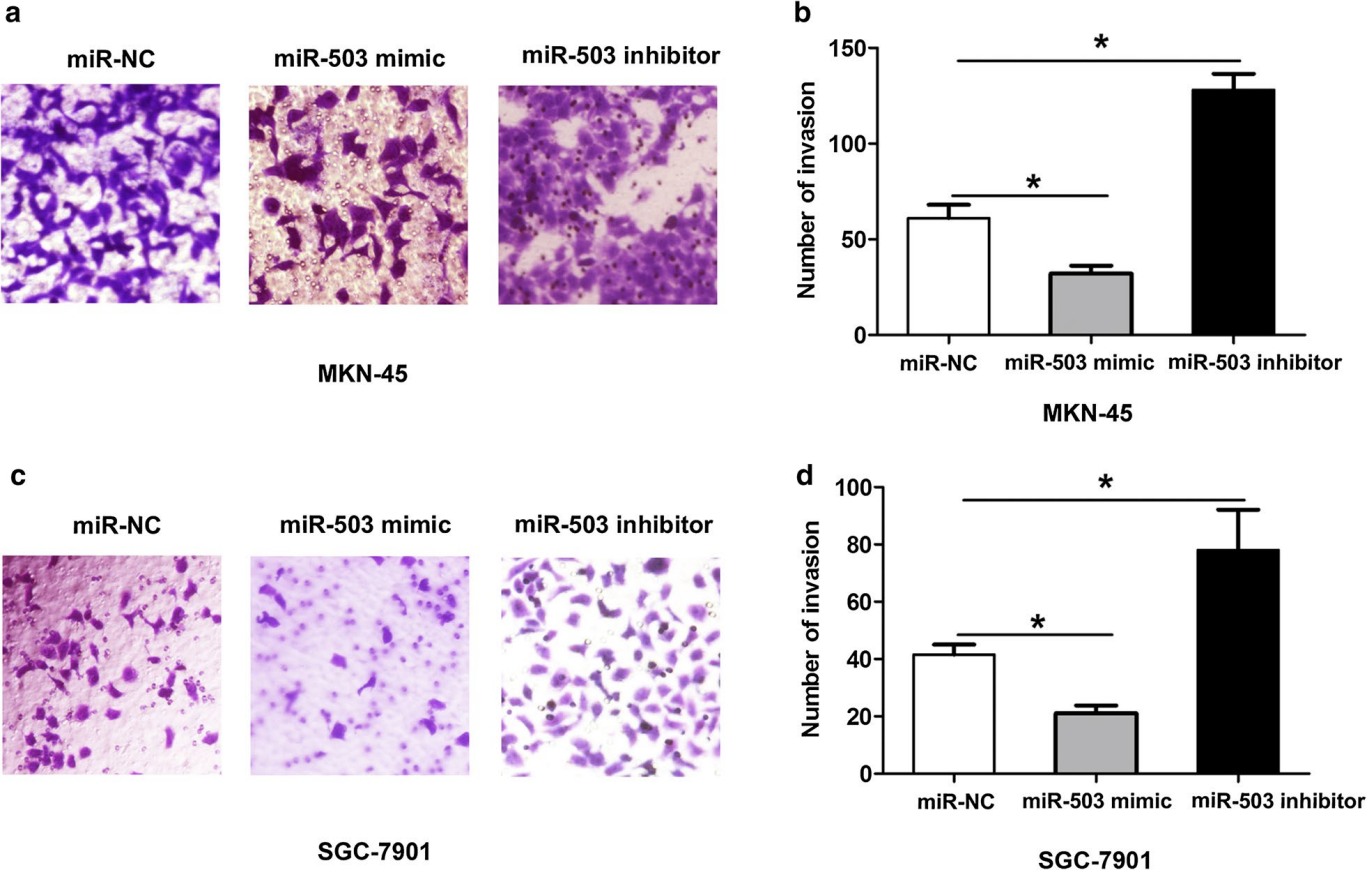
Effects of miR-503 expression on cell invasion of GC. **a**, **b** Transwell cell invasion assay and cell invasion number was showed at 48 h after cell transfected with miR-NC, miR-503 mimic or miR-503 inhibitor in MKN-45 cells. **c**, **d** Transwell cell invasion assay and cell invasion number was showed at 48 h after cell transfected with miR-NC, miR-503 mimic or miR-503 inhibitor in SGC-7901 cells. All values are presented as mean ± SD, *P < 0.05

To explore the biological functions of miR-503 in GC cells, we performed gain-function and loss-function assays. First, we applied CCK8 and cell colony formation assays to analyze the effects of miR-503 expression on cell growth of GC. CCK8 assays showed that upregulation of miR-503 significantly suppressed cell proliferation in MKN-45 and SGC-7901 cells, while downregulation of miR-503 dramatically promoted cell growth in MKN-45 and SGC-7901 cells (Fig. 2a, b). Consistently, cell colony formation assays showed that miR-503 overexpression resulted in fewer and smaller colonies. In contrast, reduced miR-503 showed adverse effects in MKN-45 and SGC-7901 cells (Fig. 2c–f). Then, we performed transwell assay to detect the effects of miR-503 on cell invasion of GC. The analysis results demonstrated that miR-503 overexpression suppressed cell invasion ability, while the inhibitor of miR-503 enhanced cell invasion ability in MKN-45 and SGC-7901 cells (Fig. 3a–d). Taken together, the above results suggested that miR-503 inhibited cell proliferation and invasion in GC cells.

### HMGA2 is a direct target of miR-503 in GC cells

**Fig. 4:**
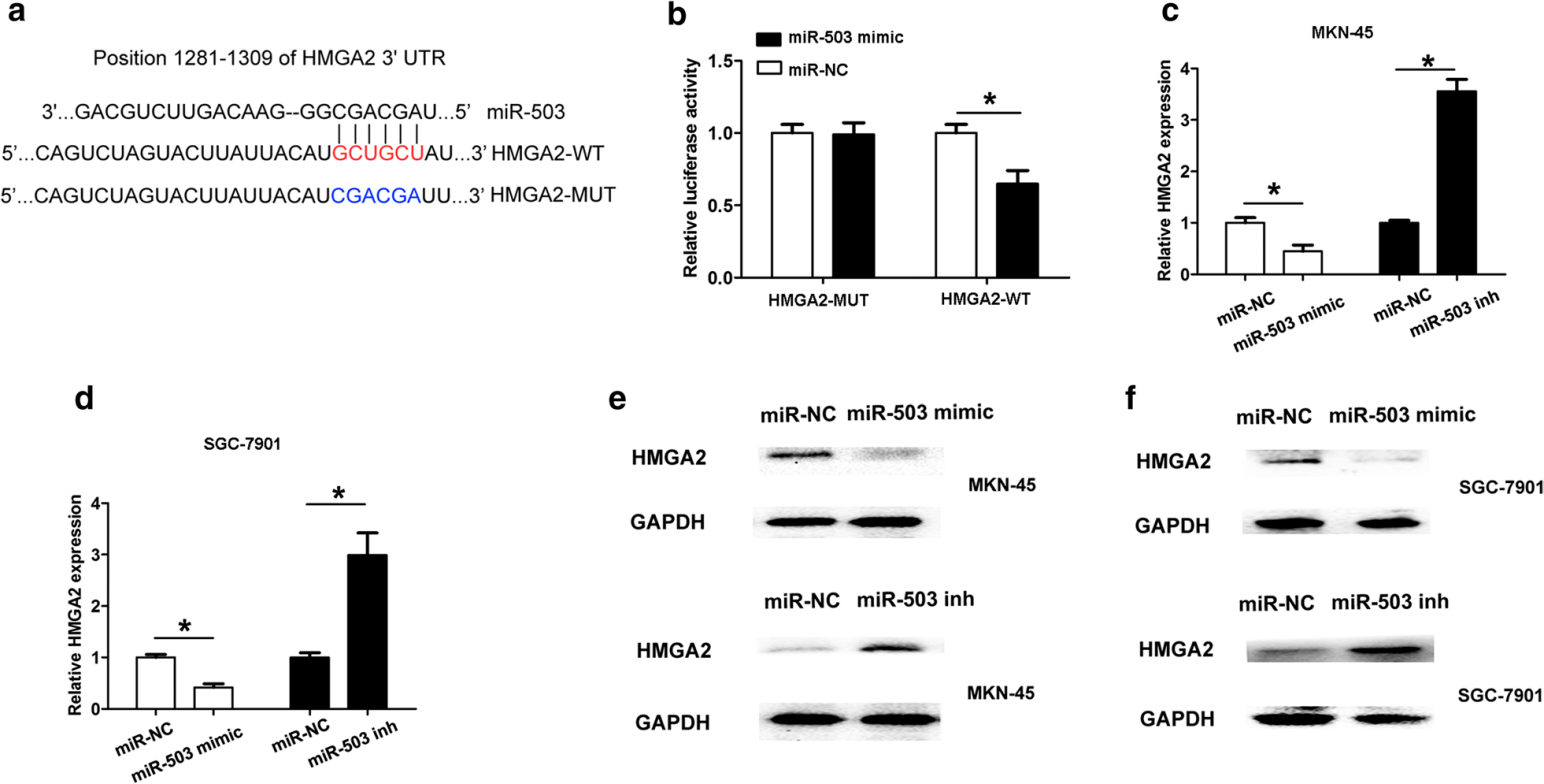
HMGA2 is a target of miR-503 in GC cells. **a** Base-pairing interaction between miR-503 seed sequences and HMGA2, as predicted by TargetScan. The wild-type (WT) HMGA2-3′UTR containing the putative miR-503 binding site or mutant HMGA2-3′UTR (MUT) (from GCUGCU to CGACGA) was cloned into the SpeI and MluI sites of the pMIR-Report luciferase vectors. **b** The luciferase activity was shown when MKN45 cells were cotransfected with pMIR-HMGA2-3′UTR-WT or pMIR-HMGA2-3′UTR-MUT and miR-503 mimic or miR-NC oligonucleotides. **c**, **d** The mRNA expression levels of HMGA2 was detected using qRT-PCR analysis after cell transfected with miR-NC, miR-503 mimic or miR-503 inhibitor in MKN-45 or SGC-7901 cells. **e**, **f** The protein expression levels of HMGA2 was detected using qRT-PCR analysis after cell transfected with miR-NC, miR-503 mimic or miR-503 inhibitor in MKN-45 or SGC-7901 cells. All values are presented as mean ± SD, *P < 0.05

**Fig. 5:**
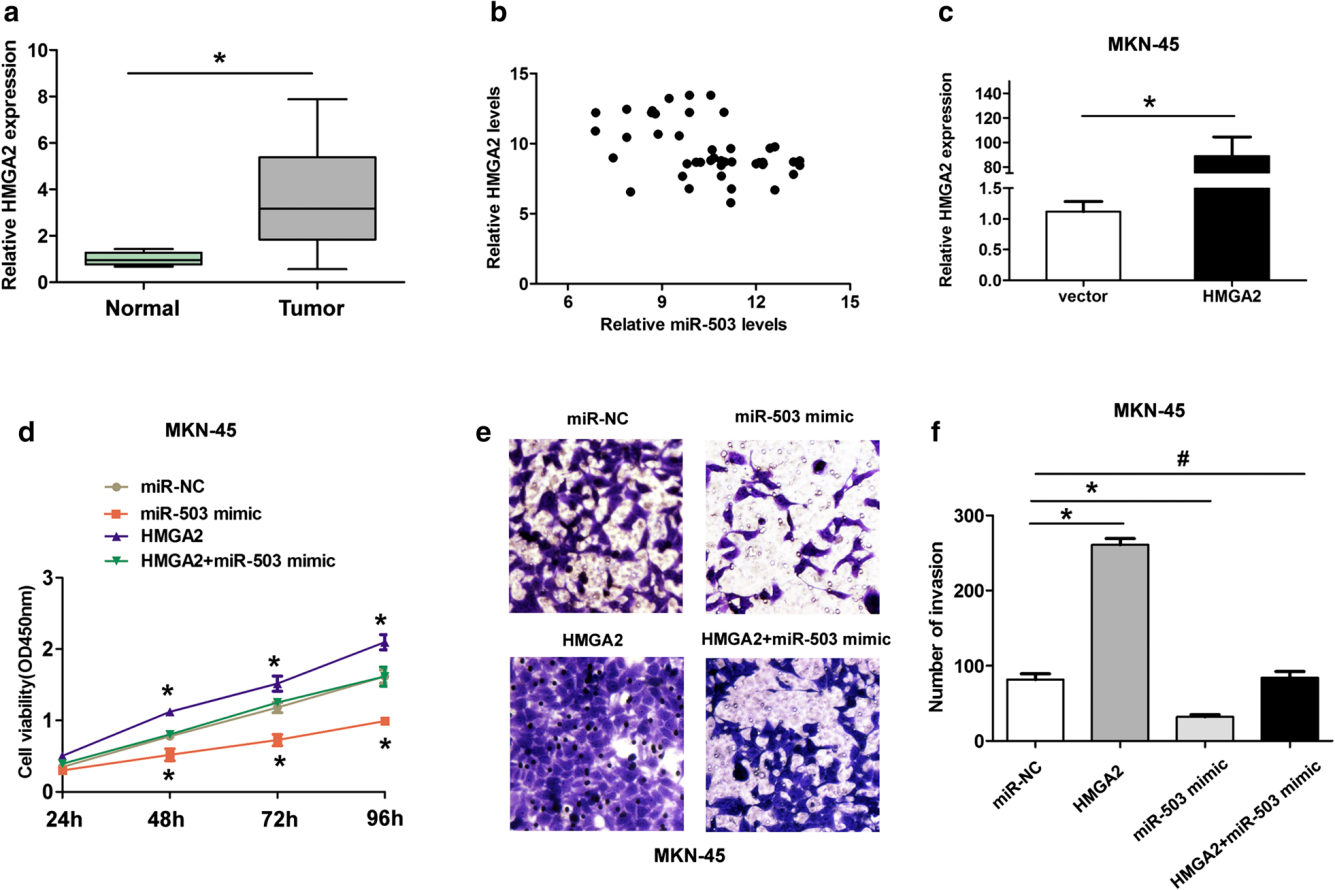
MiR-503 suppresses cell proliferation and invasion of GC cells by targeting HMGA2. **a** The expression levels of HMGA2 in 46 GC tissues and adjacent normal tissues were investigated and qRT-PCR analyses. **b** Higher HMGA2 expression levels showed a negative association with lower miR-503 in GC tissues by Spearman correlation analysis (Fig. 5b, r = -− 0.442, *P* < 0.05). **c** The expression levels of HMGA2 were detected after MKN-45 cells were trasfected with pcDNA3.1-vector or pcDNA3.1-HMGA2. **d** Cell viability was showed at 24 h, 48 h, 72 h and 96 h after cell transfected with miR-NC, miR-503 mimic, pcDNA3.1-HMGA2, or pcDNA3.1-HMGA2 + miR-503 mimic in MKN-45 cells. **e**, **f** Transwell cell invasion assay and invasive cell number was showed at 48 h after cell transfected with miR-NC, miR-503 mimic, pcDNA3.1-HMGA2, or pcDNA3.1-HMGA2 + miR-503 mimic in MKN-45 cells. All values are presented as mean ± SD, *P < 0.05, ^#^no statistical significance

According to bioinformatics analysis by online software (TargetScan, miRDB, Miranda, PicTar), HMGA2 was identified as a potential target of miR-503. The HMGA2 3′UTR fragments with wild-type (HMGA2-3′UTR-WT) or mutant (HMGA2-3′UTR-MUT) with miR-503 complementary sites were cloned into pMIR-Report luciferase vectors (Fig. 4a). MKN45 cells were co-transfected with pMIR-HMGA2-3′UTR-WT or pMIR-HMGA2-3′UTR-MUT and miR-503 mimic or miR-NC oligonucleotides. The results indicated that the luciferase activity was significantly decreased in the miR-503 mimic andpMIR-HMGA2-3′UTR-WT group, but not inmiR-503 mimic and pMIR-HMGA2-3′UTR-MUT group (Fig. 4b). These results revealed that HMGA2 was a direct target of miR-503. Furthermore, we found that transfection of miR-503 mimic suppressed the expression of HMGA2 at both the mRNA and protein levels. However, transfection of the miR-503 inhibitor increased the expression of HMGA2 in MKN-45 or SGC-7901 cells (Fig. 4c–f). Additionally, we found that HMGA2 expression levels were significantly higher in GC tissues compared to adjacent normal tissues (Fig. 5a). Higher HMGA2 expression levels showed a negative association with lower miR-503 expression in GC tissues by Spearman correlation analysis (Fig. 5b, R = − 0.442, *P* < 0.05).

### MiR-503 suppresses cell proliferation and invasion of GC cells by targeting HMGA2

We further investigated whether miR-503 suppressed cell proliferation and invasion by regulating HMGA2. MKN-45 cell was transfected with pcDNA3.1-HMGA2 to upregulated the HMGA2 expression levels (Fig. 5c). Next, CCK8 assays showed MKN-45 cells transfected with pcDNA3.1-HMGA2 significantly enhanced cell proliferation, compared with the empty vector, while the effects induced by HMGA2 overexpression was dismissed by transfecting miR-503 mimic plus pcDNA3.1-HMGA2 groups (Fig. 5d). Furthermore, MKN-45 cells transfected with pcDNA3.1-HMGA2 significantly enhanced cell invasion ability, compared with the empty vector, but the effects induced by HMGA2 overexpression was reversed by transfecting miR-503 mimic plus pcDNA3.1-SFRP1 groups (Fig. 5e, f). Therefore, these results indicated that miR-503 suppressed cell proliferation and invasion of GC cells by targeting HMGA2.

### MiR-503 overexpression inactivates the Wnt/β-catenin signaling pathway

**Fig. 6:**
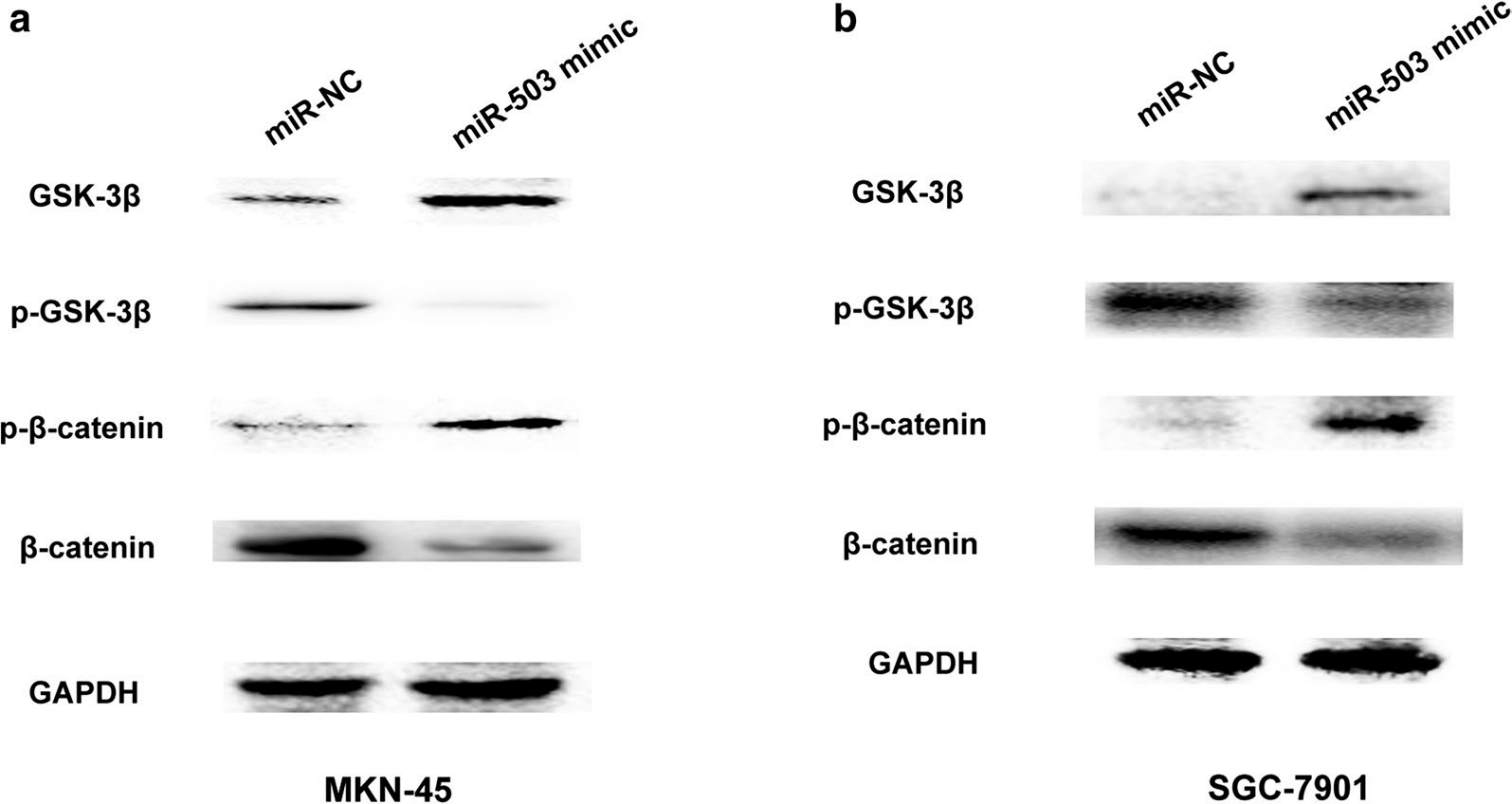
MiR-503 overexpression inactivates the Wnt/β-catenin signaling pathway. **a** The protein expression of GSK-3β, p-GSK-3β and p-β-catenin or β-catenin was showed by western blot after MKN45 cells were transfected with miR-NC or miR-503 mimic. **b** The protein expression of GSK-3β, p-GSK-3β and p-β-catenin or β-catenin was showed by western blot after SGC-7901 cells were transfected with miR-NC or miR-503 mimic

Wnt/β-catenin signaling is importantly associated with tumor proliferation and invasion [14]. We further detected whether miR-503 expression affected Wnt/β-catenin signaling. Western blot analysis results showed that miR-503 overexpression significantly elevated GSK-3β and p-β-catenin expression levels, but decreased p-GSK-3β and β-catenin expression levels in MKN-45 cells and SGC-7901 cell (Fig. 6a, b). Thus, these results indicated that miR-503 expression inactivated the Wnt/β-catenin signaling pathway in GC cells.

### MiR-503 overexpression suppresses GC cell proliferation in vivo

**Fig. 7:**
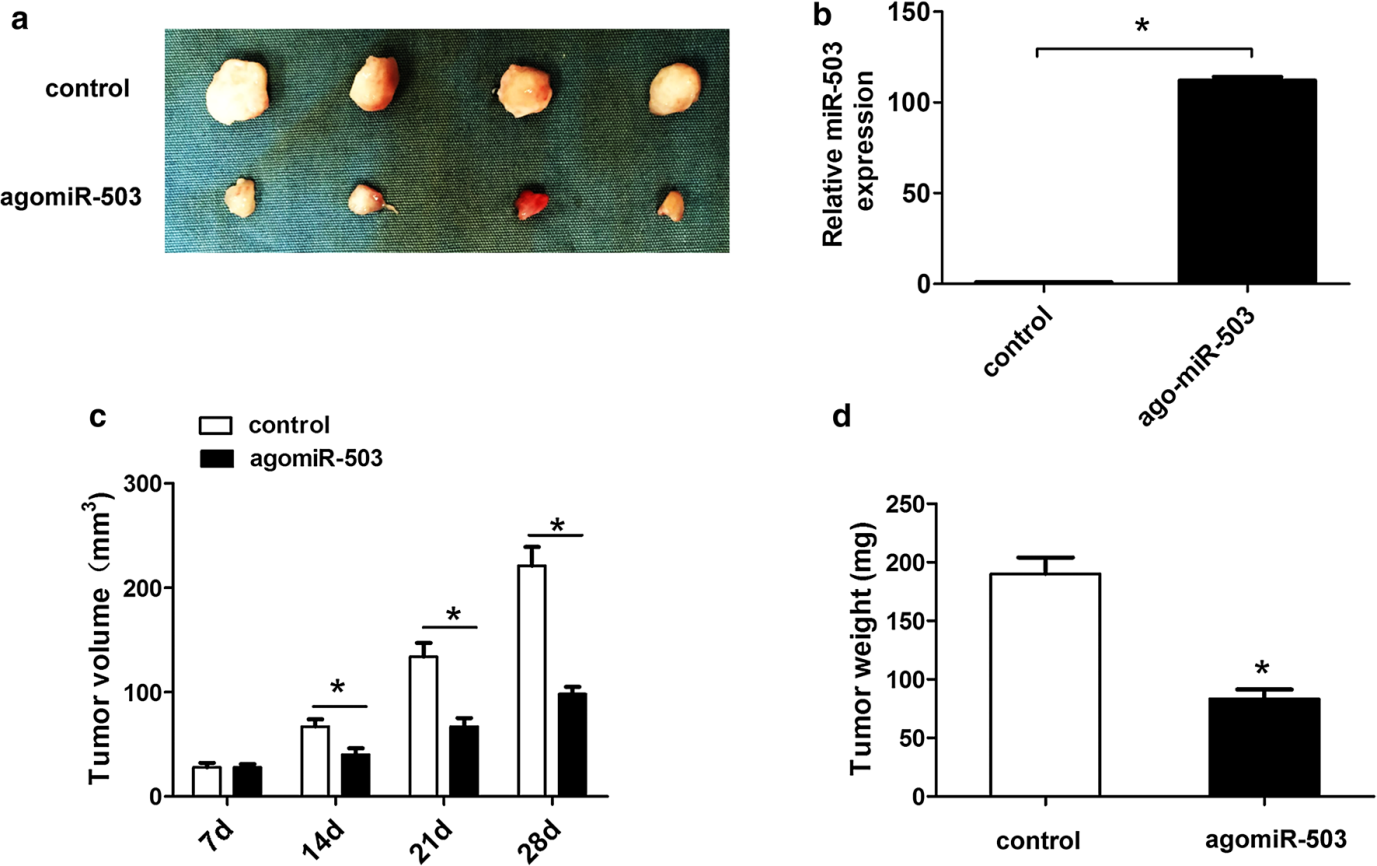
MiR-503 overexpression suppresses GC cell proliferation in vivo. **a** Tumors were harvested and immediately imaged at 28 days. **b** the miR-503 expression was detected by qRT-PCR in agomiR-control and agomiR-503 group. **c** Tumor volumes were measured every 7 days after injection using stably transfected agomiR-control and agomiR-503 plasmids. **d** Tumors were harvested and tumor weight was immediately measured at 28 days. All values are presented as mean ± SD, *P < 0.05

The BALB/c mice (female and 4 weeks old) were subcutaneously injected using stably transfected agomiR-control and agomiR-503 plasmids using MKN-45 cells (1 × 10^6^ cells) into the left flank. We observed that the tumor size was smaller in agomiR-503 group compared to the control group (Fig. 7a). Futhermore, we found that the miR-503 expression was higher in agomiR-503 group compared to the control group (Fig. 7b). Tumor volume was also smaller in agomiR-503 group, compared to the control group (Fig. 7c). Moreover, the tumor weight was also reduced in agomiR-503 group, compared to the control group (Fig. 7d). These results indicated miR-503 overexpression suppressed GC cell proliferation in vivo.

## Discussion

MiRNAs function as oncogenes or tumor-suppressor genes to be involved in tumorigenesis and cancer progression including GC [7]. Aberrant miRNA expression is identified as prognostic biomarkers and therapeutic targets for GC. MiR-503 had been reported as tumor suppressors in some cancer types. For instance, Guo et al. reported that miR-503 suppresses tumor cell proliferation and metastasis by directly targeting RNF31 in prostate cancer [15]. Overexpression of miR-503 in breast cancer cell lines reduced cell proliferation through inducing G0/G1 cell cycle arrest by targeting CCND1 [16]. Yang et al. revealed that MiR-503 targets PI3K p85 and IKK-β and suppresses progression of non-small cell lung cancer [17]. In the previous report, miR-503 was also identified as tumor suppressor in gastric cancer and can inhibit EMT in gastric cancer cells [12]. Decreased miR-503 expression in gastric cancer is inversely correlated with serum carcinoembryonic antigen and acts as a potential prognostic and diagnostic biomarker [18]. In the study, we investigated that the clinical significance and underlying molecular mechanisms for miR-503 in GC. We demonstrated that miR-503 expression was downregulated in gastric cancer tissues and cell lines, respectively. Lower miR-503 expression associated with lymph node metastasis and large tumor size of GC patients. Furthermore, lower miR-503 expression predicted a poor prognosis of GC. In vitro assays, we also demonstrated miR-503 overexpression suppressed cell proliferation and invasion in vitro. In vivo, miR-503 overexpression reduced tumor growth. These above results indicated that miR-503 suppressed the GC malignant progress in vitro and in vivo.

**Fig. 8:**
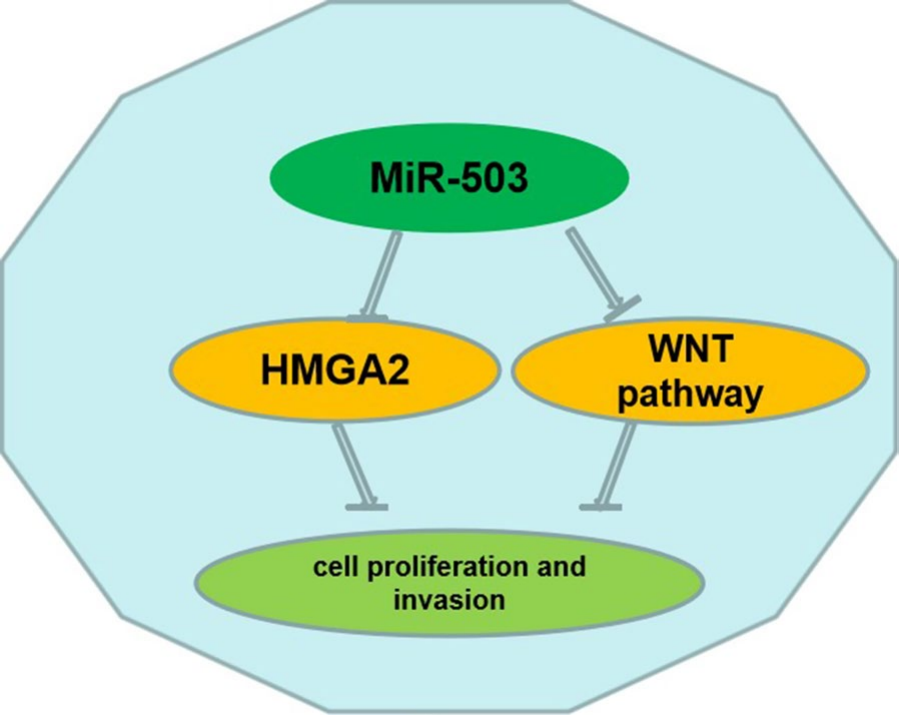
MiR-503 suppresses cell proliferation and invasion of gastric cancer by targeting HMGA2 and inactivating WNT signaling pathway

HMGA2 is a nuclear-binding protein that plays key regulators in cell growth and invasion. In gastric cancer development and progression, HMGA2 was identified as oncogenes in previous study. Elevated HMGA2 expression levels were significantly associated with poor clinical prognosis [19]. HMGA2-FOXL2 axis regulates metastases and epithelial-to-mesenchymal transition of chemo-resistant gastric cancer [20]. HMGA2 regulates epithelial–mesenchymal transition and the acquisition of tumor stem cell properties through TWIST1 in gastric cancer [21]. HMGA2 could promote vasculogenic mimicry and tumor aggressiveness by upregulating Twist1 in gastric carcinoma [22]. In GC, the other targets of miR-503 showed little report. Li showed that upregulation of circular RNA circ-ERBB2 predicts unfavorable prognosis and facilitates the progression of gastric cancer via miR-503/CACUL1 signaling [23]. Our results indicated that HMGA2 is a target of miR-503 in GC, and HMGA2 overexpression promoted GC cell proliferation and invasion. Moreover, HMGA2 overexpression mediated the inhibiting effects by miR-503 on GC cell proliferation and invasion (Fig. 8).

Next, we also demonstrated that miR-503 could inactivate WNT/β-catenin signaling pathway by elevating GSK-3β and p-β-catenin expression levels, in GC cells. In previous report, the WNT/β-catenin signaling pathway activating could promote cell proliferation and invasion of GC [24]. Such as, microRNA-140-5p suppresses cell proliferation and invasion in gastric cancer by targeting WNT1 in the WNT/β-catenin signaling pathway [25]. MIR-519d suppresses the gastric cancer epithelial–mesenchymal transition via Twist1 and inhibits Wnt/β-catenin signaling pathway [26]. In the study, our results indicated that miR-503 is a key regulator for WNT/β-catenin signaling pathway and upregulation of miR-503 inhibits Wnt/β-catenin signaling pathway (Fig. 8).

## Conclusion

Taken together, our studies confirmed that miR-503 expression was downregulated in gastric cancer and lower miR-503 expression associated with poor prognosis of patients. MiR-503 overexpression suppressed cell proliferation in vitro and in vivo. Furthermore, we demonstrated miR-503 suppressed cell proliferation and invasion through regulating HMGA2 expression and suppressed WNT/β-catenin signaling pathway. Thus, these results indicated that miR-503 may be a potential target of GC treatment.

## Data Availability

We declared that materials described in the manuscript, including all relevant raw data, will be freely available to any scientist wishing to use them for non-commercial purposes, without breaching participant confidentiality.

## Abbreviations

MiRNAs: microRNA
DMEM: Dulbecco’s Modified Eagle's Medium
FBS: fetal bovine serum
